# Prevalence of zero-sanitation in India: Patterns of change across the states and Union Territories, 1993-2021

**DOI:** 10.7189/jogh.13.04082

**Published:** 2023-07-28

**Authors:** Anoop Jain, Akhil Kumar, Rockli Kim, S V Subramanian

**Affiliations:** 1 Global Health & Social Medicine, Harvard Medical School, Boston, Massachusetts, USA; 2 Center for Geographic Analysis, Harvard University, Cambridge, Massachusetts, USA; 3 Division of Health Policy & Management, College of Health Science, Korea University; 4 Harvard Center for Population and Development Studies, Cambridge, Massachusetts, USA; 5 Department of Social and Behavioral Sciences, Harvard T.H. Chan School of Public Health, Boston, Massachusetts

## Abstract

**Background:**

Ensuring universal access to safe sanitation by 2030 is a development priority for India. Doing so can help ensure improved physical and mental health outcomes. While the proportion of people in India with safe sanitation has risen dramatically over the past thirty years, much less is known about who has been most at risk for not having access to safe sanitation across India’s states and Union Territories (UT) over this time period. We introduce the concept of zero-sanitation to fill this gap.

**Methods:**

Data from five National Family Health Surveys (NFHS) conducted in 1993, 1999, 2006, 2016, and 2021 from 36 states and UT were used for this study. The study population consisted for all household individuals regardless of age in each survey round. Zero-sanitation was defined as those who have no access to a household toilet, and thus defecate in the open. We analyzed the percent prevalence of zero-sanitation in every state / UT at each time period in both urban and rural communities, as well as the population headcount burden in 2021. We calculated the absolute change on an annual basis to assess the change in percentage points of zero-sanitation across time periods at the all-India and state / UT levels.

**Results:**

The all-India prevalence of zero-sanitation declined from 70.3% (95% confidence interval (CI) = 70.2%-70.5%) in 1993 to 17.8% (95% CI = 17.7%-17.9%) in 2021. The median percent prevalence of zero-sanitation across states and UTs was 65.9% in 1993. By 2021, the median percent prevalence of zero-sanitation across states and UTs was 5.7%. This reduction corresponded with a reduction in the between state / UT inequality in zero-sanitation. Nevertheless, as of 2021, the prevalence of zero-sanitation was still above 20% in Bihar, Jharkhand, Madhya Pradesh, Odisha, Rajasthan, and Uttar Pradesh. Additionally, as of 2021, almost 92% of individuals who were defecating in the open were experiencing zero-sanitation. Zero-sanitation remains most common in states such as Bihar, Punjab, Uttar Pradesh, and Assam. Nevertheless, at this current rate of improvement, every state and UT except for Sikkim and Chandigarh are on track to end open defecation by 2030.

**Conclusions:**

The concept of zero-sanitation is a useful tool in helping policy makers assess the extent to which sanitation coverage remains incomplete. When viewed through this lens, we see that open defecation remains most common among those who do not have a toilet. Addressing the myriad social determinants of sanitation access can help fill these gaps and ensure equitable sanitation coverage throughout India.

Sustainable Development Goal (SDG) 6.2 calls for universal access to safely managed sanitation by 2030. This is defined as improved toilets that are not shared between households and where the excreta are safely managed either in situ or offsite [1]. Ensuring access to safely managed sanitation is vital from a public health standpoint. The latest Global Burden of Disease evidence shows that inadequate access to sanitation is significantly associated with increased morbidity and mortality around the world [2]. In India, prior studies demonstrate how inadequate sanitation leads to the spread of faecal contamination in ground water [3]. In turn, this is associated with diarrhoea and repeated intestinal infections that cause malnutrition and growth failure [4,5], a significant risk factor for under-five mortality [6-9]. More recently, an emerging body of literature has emphasized the intrinsic value of sanitation [10], suggesting that there are benefits beyond physical health when people have access to improved sanitation. Inadequate access to sanitation is associated with a whole host of deleterious mental health outcomes [11-13].

However, improving access to safely managed sanitation has long been one of India’s greatest development challenges. In 1986, Indian government launched the Central Rural Sanitation Programme (CRSP). The aim of the program was to increase access to household toilets in rural areas through the provision of financial subsidies and by raising awareness about the importance of sanitation and hygiene. In 1999, CRSP evolved into the Total Sanitation Campaign (TSC), a more supply-side program that promoted toilet construction by opening sanitary marts that sold the materials needed to build toilets at a discounted rate. TSC also emphasized the construction of toilets in non-household settings, such as schools. India’s sanitation program was rebranded and rebooted once again in 2012. This time it was called Nirmal Bharat Abhiyan (NBA), a program that shifted back to a demand-generation approach that stressed the importance of toilet construction and use using information, education, and communication (IEC). In 2014, the government once again changed the name of the program to Swachh Bharat Abhiyan (Clean India Mission). However, this program kept a demand-side approach, leaning on IEC to change attitudes, knowledge, and beliefs about the importance of using toilets, and the dangers of open defecation [14]. Swachh Bharat Abhiyan (SBA) offered INR 12 000 (151 US dollars (US$) in 2022) to poor households as a further financial incentive to spur demand for toilets [14].

The World Health Organization’s Joint Monitoring Programme (JMP) tracks the progress nations are making towards improving access to safe sanitation. JMP data shows that in 1990, only 18% of India’s population were using toilets [15]. By 2011, the percent of people with a toilet almost doubled to 35% [15]. This pace of improvement increased dramatically over the past decade. By 2015, 57% of Indians had a toilet, while 29% were defecating in the open [1]. In 2020, the percent of Indians with a toilet had risen to 71% [1].

What remains unknown, however, is an understanding of who has been least likely to gain access to safe sanitation over the past thirty years across India’s states and Union Territories (UTs). We examine this by introducing the concept of zero-sanitation. We define zero-sanitation as those who have no access to a household toilet, and thus defecate in the open. Open defecation is defined as the disposal of human excrement in fields, forests, bushes, open bodies of water, or other open places [1]. Elucidating trends in zero-sanitation can help inform the design of future policies and interventions. More specifically, the results from this paper could help policy makers understand where sanitation interventions should be targeted to ensure equitable access to safe sanitation.

Therefore, the purpose of this paper is to assess sub-national trends in zero-sanitation between 1993 and 2021. Using a novel method, we conduct this analysis using present-day state / UT geometries. We also provide a headcount estimates of zero-sanitation individuals in each state and UT as of 2021. Similarly, we show the prevalence of zero-sanitation by household wealth, education, and caste in 2021. Finally, we assess which states are on track to meet Sustainable Development Goal 6.2, which calls for an end to open defecation by 2030.

## METHODS

### Data source

This is a cross-sectional analysis from five different time points. We used data from five rounds of India’s National Family Health Survey (NFHS): NFHS-1, from 1993, NFHS-2, from 1999, NFHS-3, from 2006, NFHS-4, from 2016, and NFHS-5, from 2021. Each round of the NFHS is conducted in a representative household sample. The survey has been designed to capture indicators of population health and nutrition. The NFHS, which is a Demographic and Health Survey (DHS) has a two-stage sampling process. First, Primary Sampling Units (PSUs), which are villages in rural areas and wards in urban areas, were selected with probability proportional to size from districts within states. Second, households were then randomly selected from each PSU.

### Study population

The study population were all individuals in a given household at the time of the survey, regardless of age. Observations for which household toilet access was reported as “do not know” or missing were excluded from this analysis. The percentage of excluded observations was less than 1% in every round. The final analytic sample is presented in **Table 1**.

**Table 1:** Study sample size selection from the five National Family Health Surveys, 1993-2021

	Number of respondents with complete sanitation data	Number of individuals with missing sanitation data	Final study sample size (n)
NFHS-1 (1992-1993)	492 798	0	492 798
NFHS-2 (1998-1999)	498 303	47	498 256
NFHS-3 (2005-2006)	516 251	478	515 773
NFHS-4 (2015-2016)	2 801 958	0	2 801 958
NFHS-5 (2019-2021)	2 795 894	7	2 795 887
All waves	7 105 204	532	7 104 672

NFHS – National Family Health Surveys

### Outcome definition

We used the toilet responses from across surveys to define zero-sanitation (**Table 2**). Toilet questions were asked of the de jure members of each household in the following manner in NFHS-1 and NFHS-2: “What kind of toilet facility does your household have?” and in the following manner in NFHS-3, NFHS-4, and NFHS-5: “What kind of toilet facility do members of your household usually use?” The respondent was asked to report the toilet access and use for the entire household and therefore the response of the household was applied to each household member. To construct a comparable metric of zero-sanitation across all surveys, the respondent must not enter a response of any of the options listed in **Table 2** for a particular survey year. In NFHS-1 and NFHS-2 this meant not entering a response to seven options that described toilet use, and in NFHS-3 and NFHS-4 it meant not entering a response to 11 options that described toilet use. Even though differences in toilet responses varied across surveys, the response of “Other” is consistent and is a “catch-all” for any other toilet the household may have or use.

**Table 2:** Sample size (n) and prevalence (%) of toilet items for the entire population from the five National Family Health Surveys, 1993-2021

Household toilet responses	NFHS-1, (1992-93), n = 492 798	NFHS-2, (1998-99), n = 498 256	NFHS-3, (2005-06), n = 515 773	NFHS-4, (2015-16), n = 2 801 958	NFHS-5, (2019-21), n = 2 795 887
Flush toilet					
*Own flush toilet*	97 655 (19.8%)	110 445 (22.2%)	*	*	*
*Shared flush toilet*	10 724 (2.2%)	15 438 (3.1%)	*	*	*
*Public flush toilet*	7842 (1.6%)	13 540 (2.7%)	*	*	*
Pit toilet / latrine					
*Own pit toilet / latrine*	53 792 (10.9%)	71 143 (14.3%)	*	*	*
*Shared pit toilet / latrine*	8025 (1.6%)	9316 (1.9%)	*	*	*
*Public pit toilet / latrine*	4865 (1.0%)	3223 (0.7%)	*	*	*
Flush or pour flush toilet					
*Flush to piped sewer system*	*	*	76 206 (14.8%)	172 508 (6.2%)	199 341 (7.1%)
*Flush to septic tank*	*	*	136 223 (26.4%)	914 781 (32.6%)	1 250 571 (44.7%)
*Flush to pit latrine*	*	*	36 604 (7.1%)	282 958 (10.1%)	390 317 (13.9%)
*Flush to somewhere else*	*	*	15 161 (2.9%)	27 013 (0.9%)	21 218 (0.8%)
*Flush, don't know where*	*	*	701 (0.1%)	3364 (0.1%)	3812 (0.1%)
Pit latrine					
*Ventilated improved single*† *pit (vip) / biogas latrine*	*	*	1641 (0.3%)	20 768 (0.7%)	18 535 (0.7%)
*Single*† *pit latrine with slab*	*	*	21 876 (4.2%)	170 061 (6.1%)	176 427 (6.3%)
*Single*† *pit latrine without slab / open pit*	*	*	19 537 (3.8%)	78 996 (2.8%)	42 898 (1.5%)
Other toilets					
*Twin pit / composting toilet*	*	*	732 (0.1%)	7420 (0.3%)	143 858 (5.2%)
*Dry toilet*	*	*	5036 (0.9%)	32 304 (1.2%)	36 988 (1.3%)
*Other*	338 (0.07%)	555 (0.1%)	1401 (0.3%)	4829 (0.2%)	8325 (0.3%)
Household members do not use/have any of the above toilets					
*Do members of your household have access to a toilet facility? | Response: No*	*	*	*	*	462 815 (91.9%)
*Zero-sanitation*	309 557 (62.8%)	274 596 (55.1%)	200 655 (38.9%)	1 086 956 (38.8%)	462 815 (16.6%)

*Represents toilet items not asked in the respective survey. For each survey, zero-sanitation was calculated separately based on the toilet items asked in the respective survey and includes respondents who did not have or use any of the listed toilet items above.

†Single only exists in NFHS-5. The question asked in NFHS-1 and NFHS-2 was: “What kind of toilet facility does your household have?” and the question asked in NFHS-3, NFHS-4, and NFHS-5 was: “What kind of toilet facility do members of your household usually use?”

Based on this, we define the prevalence of zero-sanitation among the total population as following:

zero-sanitation population / number of total people x 100

Where, the zero-sanitation population represents the total population living in households that do not have or use a toilet. As a sensitivity, NFHS-5 asked an additional question on household toilet access for household respondents who did not use a toilet. It was asked in the following manner if the household respondent said the household members usually did not use a toilet: “Do members of your household have access to a toilet facility?” Based on a response of “No” to this question, we define the prevalence of zero-sanitation in NFHS-5.

### Constructing comparable state estimates

There are currently 28 states and eight UTs in India, however the geometry of states / UTs have changed significantly over time. For example, in 1993, there were only 25 states and seven UTs in India and these changes mean that creating a repeated cross-sectional panel of the states / UTs of India required making the states comparable over time. Unlike popular approaches of aggregating the latest geometry into older state-geometries, we employed a new method that assigned districts surveyed in earlier years to current day states [16]. An example of this is the states of Madhya Pradesh and Chhattisgarh, which in NFHS-1 and NFHS-2 were only represented as one state (Madhya Pradesh). Using the method described in Subramanian et al. [16], we were able to align the geometry to the current states / UTs to help with current policy deliberations happening on the latest geography. These estimates were derived using the mean command in Stata 17 with the default option of reporting the 95% confidence intervals.

### Demographic and socioeconomic correlates

We also examined patterns of zero-sanitation by household wealth quintile (lowest, low, middle, high, highest), household caste (Other, Other Backwards Caste, Scheduled Caste, and Scheduled Tribe), and highest level of educational attainment (no school, primary, secondary, and above 12^th^ grade). We estimated the unweighted mean of zero-sanitation by each marker of socioeconomic status and present the 95% confidence intervals (CIs).

### Analysis

To estimate trends for India and states / UTs over time, we calculated the prevalence (and 95% CIs) of zero-sanitation for India and each state / UT as each time period (1993, 1999, 2006, 2016, and 2021). The prevalence estimates made use of DHS survey weights to account for the survey design in each survey. To assess the change in state-inequalities in zero-sanitation over time, we used boxplots to show the distribution of the prevalence of zero-sanitation across states for each time period.

To assess which states are on target to meet the SDG 6.2 goal of becoming open defecation free, we used the zero-sanitation values to calculate the SDG status for India and each state / UT. We first calculated the actual annual absolute change in zero-sanitation between 2016 and 2021. We did this by using equation 1 below



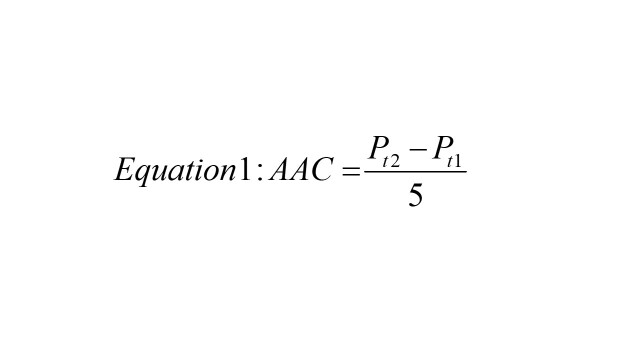



where *P_t2_* is the prevalence in 2021 and *P_t1_* is the prevalence in 2016. Next, we calculated the required annual changed needed to achieve SDG 6.2 using equation 2 below



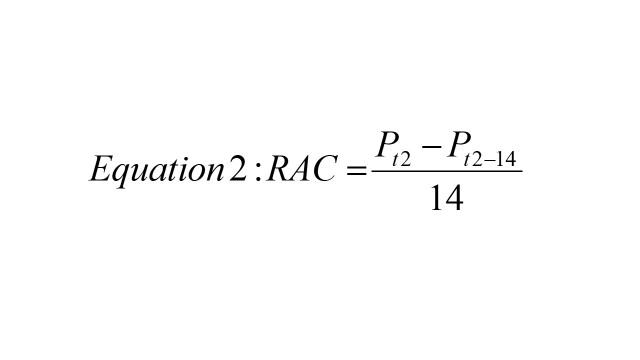



where *P_t2_* is the 2030 target (0% open defecation) and *P_t2-14_* is the prevalence in 2016. For any given state, if AAC is less than RAC, we can say that the state is on target for the 2030 goal as the actual rate of change is faster than the required rate of change. Then, we estimate the predicted year when each state will reach this target by using equation 3 below



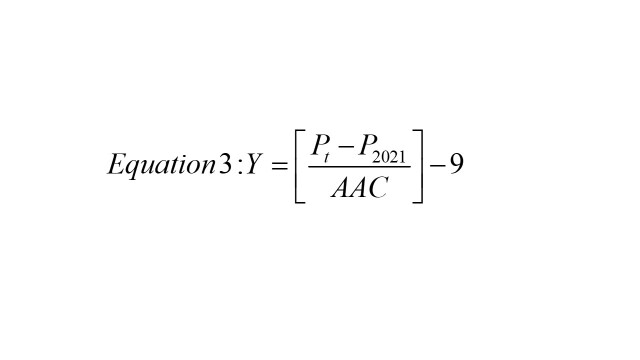



Where *Y* is the number of years required after 2030 to meet the target, *P_t_* is the 2030 target, and *P_2021_* is the prevalence in 2021. The methodology and application of equations 1-3 has been validated in previously published work [17].

We estimated the absolute burden (population headcount) of zero-sanitation in 2021 for all India and for each state / UT. This was done by combining the microdata and Census of India Population Projections [18]. We used the total population for 2021 for all age groups. We replicated the Integrated Public Use Microdata Series (IPUMS) methodology assuming a total population of 1 363 006 000 for India in 2021 [19]. This entire approach has been validated in previously published work [16].

### Ethics statement

NFHS data collection was approved by the International Institute for Population Studies Institutional Review Board (IRB) [20]. This analysis did not meet the regulatory definition of human subject research as per the Harvard Longwood Campus IRB and was thus exempt from a full IRB review.

## RESULTS

### Sample characteristics

There were 2 795 887 total de iure individuals in the NFHS-5 sample, ranging from less than one month in age to 98 years. Approximately 75% of respondents lived in rural areas. Nearly 20.5% were from Scheduled Caste households, 19.5% from Scheduled Tribe households, and 38.9% from Other Backwards Caste households. These trends were consistent over the five survey rounds. The NFHS-3 has the highest number of missing data for the type of toilet used by the household. All other survey rounds had 51 or fewer missing responses. The final analytic sample for each survey round is shown in **Table 1**. The analytic sample by type of sanitation is shown in **Table 2**.

### Patterns of change in the prevalence of zero-sanitation

**Figure 1:**
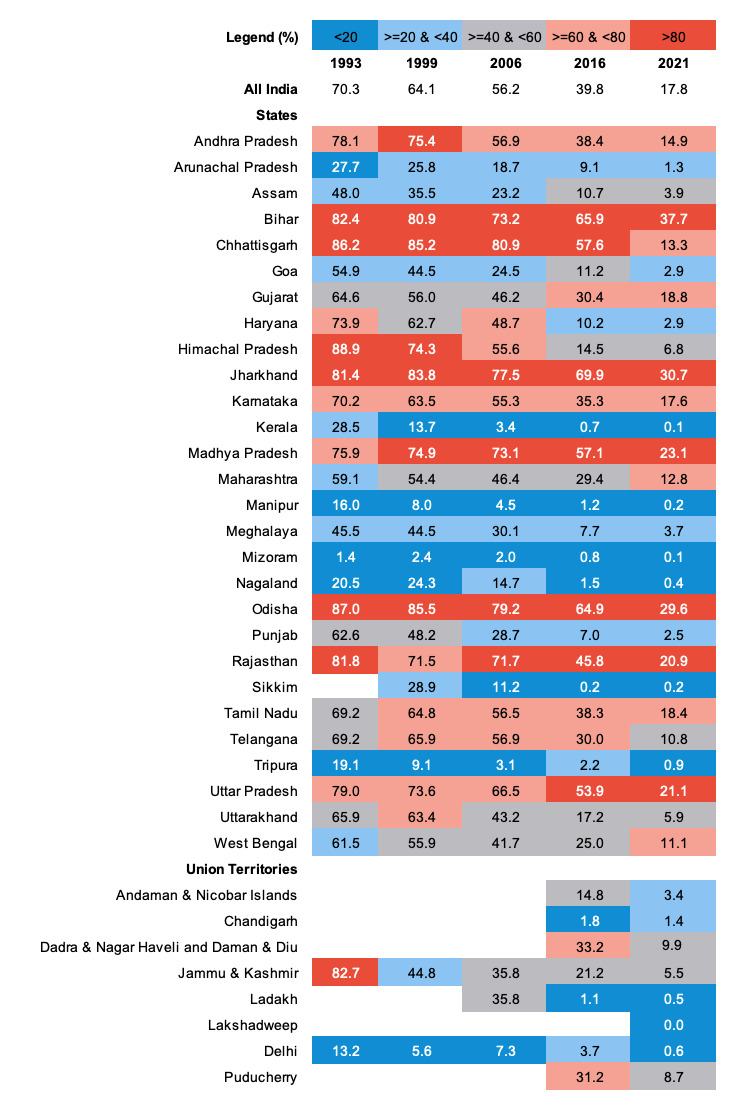
The prevalence of zero-sanitation for India and 36 states / Union Territories, 1993-2021.

**Figure 2:**
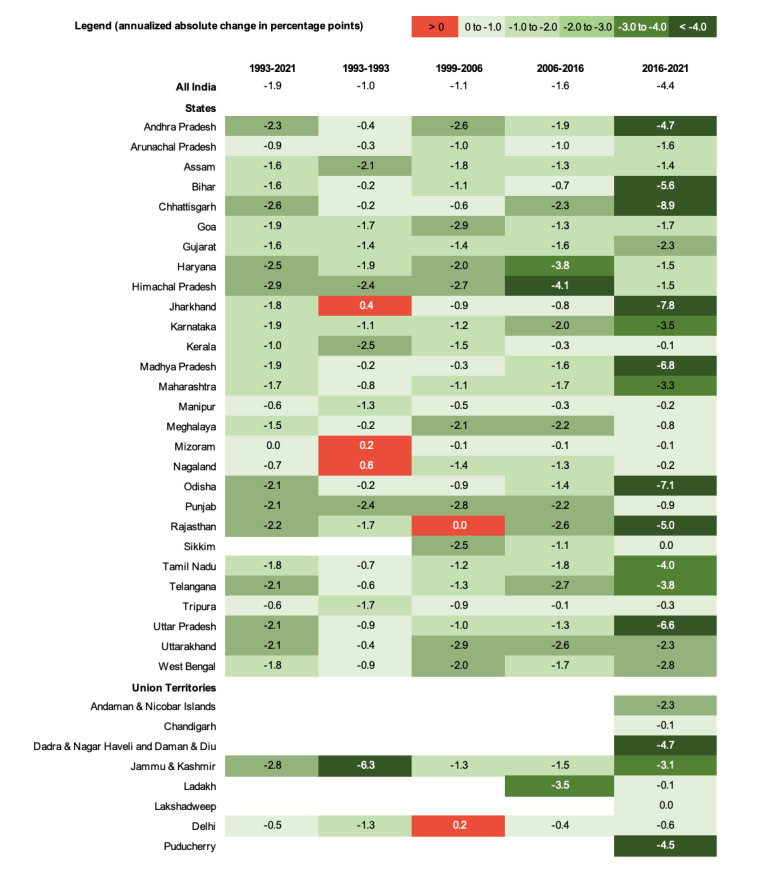
Annual absolute change (percentage points) in the prevalence of zero-sanitation across states / Union Territories of India between 1993-1999, 1999-2006, 2006-2016, 2016-2021, and 1993-2021. The legend shows the cutoff values for these changes, with darker green boxes indicating greater change. The red boxes highlight increases in zero-sanitation for a given time period in a given state / Union Territory.

In 1993, approximately 70.3% (95% CI = 70.2-70.5) of individuals in India experienced zero-sanitation. By 1999, that number had fallen by six percentage points to approximately 64.1% (95% CI = 63.9-64.2) after an annual absolute reduction of one percentage point per year. In 2006, approximately 56.2% (95% CI = 56.0-56.3) of individuals throughout India were experiencing zero-sanitation after an annual absolute reduction of 1.1 percentage points per year. By 2016, that number had decreased to approximately 39.8% (95% CI = 39.8-39.9) after an annual absolute reduction of 1.6 percentage points per year. Finally, by 2021, approximately 17.8% (95% CI = 17.7-17.9) of people throughout India were experiencing zero-sanitation after an annual absolute reduction of 4.4 percentage points per year. Additionally, we used the annual absolute change values to show that every state / UT except for Sikkim and Chandigarh are on track to meet SDG 6.2 by 2030. These results are shown in **Figure 1**, **Figure 2**, and Table S1 and Table S2 in the **Online Supplementary Document**.

In 1993, the percent prevalence of zero-sanitation in urban communities was above 10% in every state except Assam (7.2%, 95% CI = 5.9-8.5), Manipur (5.3%, 95% CI = 3.1-7.6), Meghalaya (3.3%, 95% CI = 1.1-5.6), Mizoram (0.4%, 95% CI = -0.4,1.3), Nagaland (5.2%, 95% CI = 1.7-8.7), and Tripura (1.1%, 95% CI = -0.1,2.2). As of 2021, the percent prevalence of zero-sanitation in urban communities was above 10% in only two states. These were Bihar (11.2%, 95% CI = 10.9-11.5) and Odisha (16.9%, 95% CI = 16.4-17.6). The percent prevalence of zero-sanitation in urban communities was 10% or less in all other states and UTs in 2021. These results are presented in Table S3 in the **Online Supplementary Document**.

In 1993, the percent prevalence of zero-sanitation in rural communities was at or above 90% in several states and UTs. These were Andhra Pradesh (91.4%, 95% CI = 91.0-91.9), Bihar (91.3%, 95% CI = 90.9-91.5), Chhattisgarh (97.4%, 95% CI = 97.0-97.7), Haryana (90%, 95% CI = 89.4-90.7), Himachal Pradesh (95.4%, 95% CI = 94.6-96.2), Jharkhand (92.2%, 95% CI = 91.5-92.8), Karnataka (90.8%, 95% CI = 90.4-91.2), Madhya Pradesh (93.3%, 95% CI = 93.0-93.7), Odisha (94.2%, 95% CI = 93.8-94.6), Rajasthan (93%, 95% CI = 92.7-93.3), Tamil Nadu (91.0%, 95% CI = 90.6-91.4), Telangana (92.9%, 95% CI = 92.4-93.5), Uttar Pradesh (92.8%, 95% CI = 92.6-93.0), Uttarakhand (91.5%, 95% CI = 90.6-92.5), and Jammu & Kashmir (94.6%, 95% CI = 93.5-95.6). In 2021, the percent prevalence of zero-sanitation in rural communities was above 20% in 12 states. These were Andhra Pradesh (20.1%, 95% CI = 19.8-20.4), Bihar (42.7%, 95% CI = 42.5-42.9), Gujarat (30.1%, 95% CI = 29.8-30.5), Jharkhand (37.3%, 95% CI = 36.9-37.7), Karnataka (24.4%, 95% CI = 24.1-24.6), Madhya Pradesh (29.4%, 95% CI = 29.1-29.7), Maharashtra (20.5%, 95% CI = 20.3-20.7), Odisha (32.3%, 95% CI = 32.0-32.7), Rajasthan (26.7%, 95% CI = 26.4-26.9), Tamil Nadu (28.6%, 95% CI = 28.3-28.9), Uttar Pradesh (26.4%, 95% CI = 26.2-26.5), and Puducherry (20.9%, 95% CI = 18.0-23.8). The percent prevalence of open defecation in rural communities in 2021 was below 20% in all other states and UTs. These results are presented in Table S4 in the **Online Supplementary Document**.

### Changes in the geographic distribution of the prevalence of zero-sanitation

**Figure 3:**
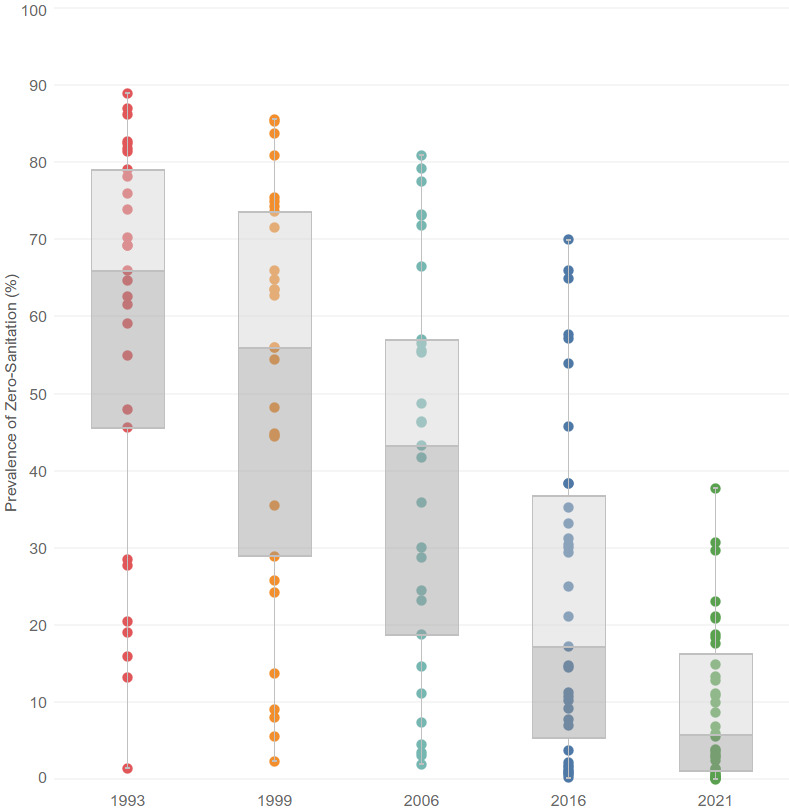
Summary distribution of state / Union Territories-level prevalence of zero-sanitation, 1993-2021. Box-and-whisker plot shows the variability of a data set using the lowest and highest values, and quartiles of the data. The upper and lower whiskers represent minimum and maximum values, respectively. The upper outline of the box depicts the 75th percentile and the lower outline the 25th percentile, respectively. The solid line within the box shows the 50th percentile (median).

Overall, the percent prevalence of zero-sanitation across states and UTs declined between 1993 and 2021. In 1993, the median percent prevalence of zero-sanitation across states and UTs was 65.9%. By 2021, this value had decreased to 5.7%. We found that the between-state / UT inequality decreased over this time. This is demonstrated by the reduction in the interquartile range (IQR), which decreased from 32.6 percentage points (25^th^ percentile = 46.1%, 75^th^ percentile = 78.7%) in 1993 to 16.3percentage points (25^th^ percentile = 1.3%, 75^th^ percentile = 17.6%) in 2021. The IQR was the highest in 1999 with a value of 40.3% (25^th^ percentile = 32.2%, 75^th^ percentile = 72.6%). These values and trends are shown in **Figure 3**

In 2021, there were several states in which the prevalence of zero-sanitation was higher than the national average. These were Bihar (37.7%, 95% CI = 37.5-37.9), Gujarat (18.8%, 95% CI = 18.6-18.9), Jharkhand (30.7%, 95% CI = 30.3-30.9), Madhya Pradesh (23.1%, 95% CI = 22.9-23.3), Odisha (29.6%, 95% CI = 29.3-29.9), Rajasthan (20.9%, 95% CI = 20.7-21.1), Tamil Nadu (18.4%, 95% CI = 18.2-18.6), and Uttar Pradesh (21.1%, 95% CI = 20.9-21.2). The percent prevalence of zero-sanitation fell below 1% in Kerala, Manipur, Mizoram, Nagaland, Delhi, and Tripura between 1993 and 2021. The percent prevalence of Zero-sanitation was below 1% in Sikkim, Ladakh, and Lakshadweep in 2021, however there is no data for these areas from 1993. These results are presented in **Figure 1** and Table S1 in the **Online Supplementary Document**.

The largest decline in prevalence of zero-sanitation between 1993 and 2021 occurred in Himachal Pradesh 82.1 percentage point reduction. The prevalence of open defecation fell by more than 70 percentage points in Haryana (71 percentage points), Chhattisgarh (72.9 percentage points), and Jammu & Kashmir (77.2 percentage points) between 1993 and 2021. These results are presented in in **Figure 1** and Table S1 in the **Online Supplementary Document**.

We produced visualizations of these state / UT-level change in Zero-sanitation between 1993 and 2021. These visuals include choropleth maps which can be seen on an interactive online dashboard (https://geographicinsights.iq.harvard.edu/State-Zero-Sanitation).

### Estimated headcount of zero-sanitation individuals in India

Overall, we found that there are approximately 238 756 244 people in India who were experiencing zero-sanitation on a daily basis in 2021. Approximately 90% of these people live in just 11 states. These are Uttar Pradesh (20.0%), Bihar (19.2%), Maharashtra (6.7%), Madhya Pradesh (7.9%), Rajasthan (7.0%), Tamil Nadu (5.9%), Odisha (5.3%), Gujarat (5.1%), Karnataka (5.1%), West Bengal (4.7%), and Jharkhand (4.4%). Lakshadweep is the only state / UT with zero people defecating in the open as of 2021. These results are presented in **Table 3**.

**Table 3:** Estimated headcount (n) of zero-sanitation individuals for India and 36 states / Union Territories (UT), and percentage share of zero-sanitation individuals of each state / UT, 2021

	Headcount	Percent of total
All India*	238 756 244	
**States**		
Uttar Pradesh	47 869 064	20.0%
Bihar	45 909 468	19.2%
Madhya Pradesh	18 826 209	7.9%
Rajasthan	16 735 434	7.0%
Maharashtra	15 984 494	6.7%
Tamil Nadu	13 973 017	5.9%
Odisha	12 721 977	5.3%
Gujarat	12 116 326	5.1%
Karnataka	12 159 792	5.1%
West Bengal	11 218 805	4.7%
Jharkhand	10 448 376	4.4%
Andhra Pradesh	7 590 754	3.2%
Chhattisgarh	4 226 208	1.8%
Telangana	3 572 744	1.5%
Assam	1 391 677	0.6%
Haryana	824 838	0.3%
Punjab	738 956	0.3%
Uttarakhand	674 125	0.3%
Jammu & Kashmir	674 121	0.3%
Himachal Pradesh	500 463	0.2%
Delhi	127 650	0.1%
Meghalaya	127 038	0.1%
Puducherry	106 113	0.0%
Kerala	36 978	0.0%
Goa	56 153	0.0%
Dadra & Nagar Haveli and Daman & Diu	51 593	0.0%
Tripura	38 031	0.0%
Chandigarh	15 277	0.0%

*The All India value is the sum of the headcount values from each state.

### Correlates of zero-sanitation

Overall, we found that zero-sanitation is most common among those in the lowest socioeconomic groups. Over 65% of those experiencing zero-sanitation in 2021 were in the lowest (43.5%, 95% CI = 43.4-43.7) and low (22.9%, 95% CI = 22.8-23.0) wealth quintiles. Additionally, approximately 22.5% (95% CI = 22.4-22.6) of those who were defecating in the open in India in 2021 belonged to a Scheduled Caste, while 20.4% (95% CI = 20.3-20.5) belonged to a Scheduled Tribe. Approximately 24.4% (95% CI = 24.3-24.5) of those defecating in the open in 2021 were from a household in which the highest level of educational attainment was no education (preschool). Similarly, approximately 19.0% (95% CI = 18.9-19.1) of those defecating in the open in 2021 were from a household in which the highest level of educational attainment was primary school. These results are presented in **Table 4**.

**Table 4:** Sample distribution and percentage (and 95% confidence interval (CI)) of zero-sanitation individuals by socioeconomic characteristics, 2021, India

	Sample distribution		Prevalence of zero-sanitation
	**n**	**Percent**	**Percent (95% CI)**
Household wealth			
*Lowest*	626 151	22.4%	43.5% (43.4-43.7)
*Low*	617 245	22.9%	22.9% (22.8-23.0)
*Middle*	565 628	20.2%	9.3% (9.2-9.4)
*High*	515 843	18.5%	1.6% (1.6-1.7)
*Highest*	471 027	16.8%	0.07% (0.06-0.08)
Household caste			
*Scheduled caste*	548 368	20.5%	22.5% (22.4-22.6)
*Scheduled tribe*	525 923	19.7%	20.4% (20.3-20.5)
*Other backwards caste*	1 040 682	38.9%	18.2% (18.1-18.3)
*None*	542 227	20.3%	7.1% (7.0-7.2)
Highest educational level attained			
*No education, preschool*	794 533	28.4%	24.4% (24.3-24.5)
*Primary*	635 946	22.8%	19.0% (18.9-19.1)
*Secondary*	1 096 682	39.2%	12.9% (12.9-13.1)
*Higher*	267 303	9.6%	4.7% (4.6-4.8)

### Empirical assessment of lack of toilet among those who open defecate

**Figure 4:**
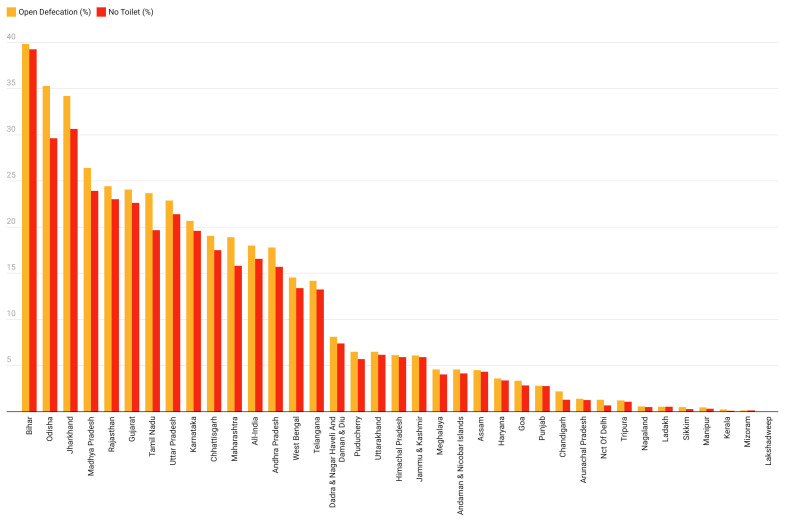
Percentage of open defecation and no toilet access in 36 states / Union Territories, 2021.

**Figure 5:**
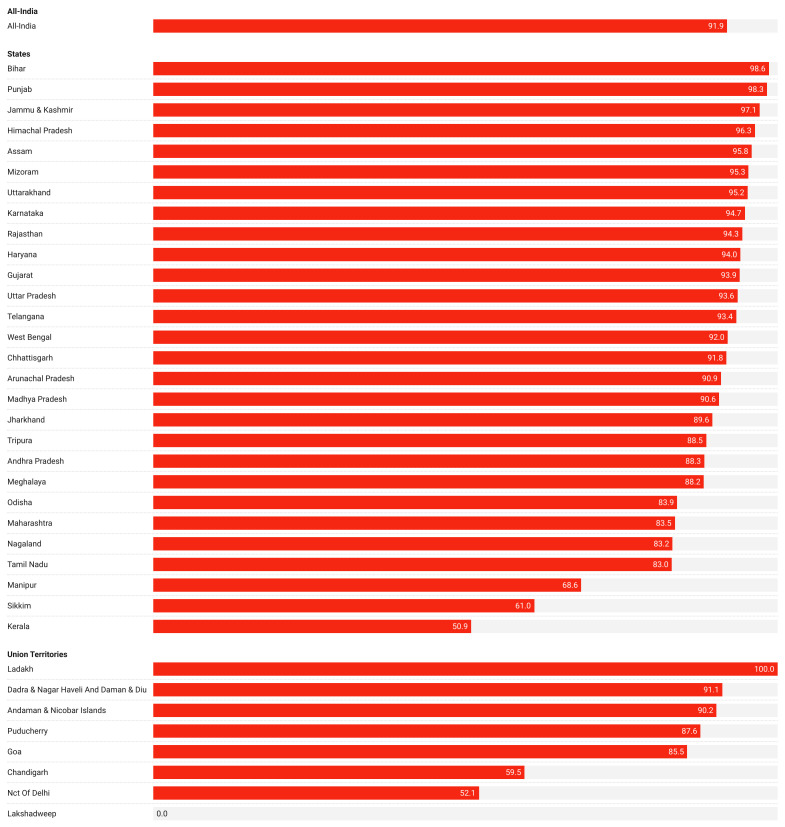
Percentage of no toilets among those who open defecate in 36 states / Union Territories, 2021.

We also found that of those defecating in the open in 2021 91.9% did not have access to a toilet, as presented in **Table 2**. Additionally, over 90% of those defecating in the open did not have a toilet in 20 states / UTs in 2021. Ladakh (100%), Bihar (98.6%), Punjab (98.2%), Himachal Pradesh (96.2%), Uttarakhand (95.2%), and Uttar Pradesh (93.6%) were among the states / UTs with the highest degree of zero-sanitation. Kerala (50.5%), Delhi (52.4%), and Sikkim (61%) were among the states / UTs with the lowest degree of zero-sanitation. These results are presented in **Figure 4** and **Figure 5**.

## DISCUSSION

This paper had four salient findings. First, we found that the median prevalence of zero-sanitation at the state / UT level fell from 65.9% in 1993 to 5.7% in 2021. This corresponded with a decline in between-state / UT inequality in zero-sanitation. Himachal Pradesh, Haryana, Chhattisgarh, and Jammu & Kashmir experienced the greatest declines over this time period. Second, we found that nearly 92% of people who were defecating in the open in 2021 did not have access to a toilet. Ladakh, Bihar, Punjab, Himachal Pradesh, Uttarakhand, and Uttar Pradesh were among the states / UTs with the highest degree of zero-sanitation in 2021. Third, we found that we found that as of 2021, zero-sanitation is most prevalent among those living in rural communities and those in the lowest socioeconomic brackets of wealth, education, and caste. Fourth, we found that at the current rate of decline in open defecation, every state / UT except for Sikkim and Chandigarh will meet the SDG 6.2 goal of eliminating open defecation by 2030.

There are three data limitations with our study. First, the NFHS was not designed to account for changes in state boundaries. Thus, the results presented in this paper might not be fully representative at the state level. Second, social desirability bias has been noted as a possible explanation for why open defecation continues being underreported throughout India [21]. This could influence the reliability of the estimates being presented in this study. Third, zero-sanitation estimates from NFHS-3 and NFHS-4 are based on the open defecation prevalence values from those two rounds. However, our results from 2021 showing that 92% of those who defecate in the open do not have access to a toilet validates this approach as this percentage was likely higher in previous years before the implementation of Swachh Bharat Abhiyan.

Examining sanitation access through the lens of zero-sanitation is important for several reasons. For instance, a prior study showed that in Bihar, Haryana, Madhya Pradesh, Rajasthan, and Uttar Pradesh, over 40% of households from the study sample had at least one member who continued defecating in the open despite the household owning a functioning toilet [22]. Another study found that in India, demand for toilets is lower than that for other consumer goods [23]. These results reinforce the conceptualization of open defecation as a behavioural issue rooted in cultural and religious notions of purity and pollution [22,24-28]. As a result, while government programmes have provided household subsidies as a way to increase sanitation supply, these programmes have also focused heavily on investing in information, education, and communication (IEC) campaigns as a way to spur demand for sanitation [14]. This demand-side approach has been reinforced by interventions such as Community-Led Total Sanitation (CLTS) that aim to increase demand for sanitation through methods rooted in triggering and shaming [29].

However, studies measuring the effects of demand-side programs have shown modest results at best. One study measured the effect of India’s Total Sanitation Campaign on open defecation in Madhya Pradesh and found that the program had very modest results in improving household toilet coverage and in reducing rates of open defecation [30]. And another study aimed at assessing CLTS found that the effectiveness of the behaviour change program is weak [31]. These behaviour change programs have failed in part because many households cannot afford the upfront cost of toilet construction [32,33], and because they are unable to address deeply entrenched social and gender hierarchies [28,34]. And people might continue defecating in the open despite toilet access because they are afraid of their pit latrines filling up too quickly [35], or because they do not have access to clean toilets in public spaces [13], an issue not rooted in behavioural choices.

Our headcount results show that over 70% of those who defecate in the open throughout India live in Bihar, Uttar Pradesh, Madhya Pradesh, Rajasthan, Gujarat, West Bengal, Assam, Chhattisgarh, and Jharkhand. The degree of Zero-Sanitation was approximately 90% or higher in each of these states as of 2021, demonstrating that access to sanitation remains very incomplete in many parts of India.

These results underscore the importance of addressing the social determinants of sanitation access. For example, those living in rural areas are less likely to have access to safe sanitation than those in urban communities given the low government prioritization of these areas [32]. Other studies have shown how progress towards building household toilets has slowed in rural India [36], which could be due to a mix of factors such as gender, age, education of the household head, and climate change [37-39]. Other location-based characteristics, such as access to water, for flushing and self-cleaning, and soil type are also important predictors of toilet ownership and use in India [40-42], and must be considered as states design and implement sanitation interventions. Dwelling space, an indicator of household wealth, is another important determinant of toilet access, and thus open defecation, in both urban and rural areas. Many poor rural and urban residents do not have space for a private toilet [43-45]. Our results showing that those belonging to India’s lowest castes are most likely to experience zero-sanitation highlights why state governments need to also address the various dimensions of caste discrimination, which remain important determinants of inadequate sanitation access [46-50].

Additionally, we show that almost every state / UT is on track to meet the SDG 6.2 goal of eliminating open defecation by 2030. However, addressing these place-based and socioeconomic determinants of open defecation are necessary to ensure that all states meet SDG 6.2, even those that are currently on target from slipping backwards [31,51,52].

## CONCLUSIONS

This paper presents the first ever prevalence estimates zero-sanitation across all 36 states / UTs within India over a 30-year period. While the overall prevalence of zero-sanitation has reduced at the state / UT level over the past 30 years, we show that states such as Bihar, Uttar Pradesh, Odisha, and Madhya Pradesh have the highest burden of zero-sanitation both in terms of prevalence and head count. We show that zero-sanitation is clustered in rural areas among the poorest and least educated households. States need to continue address the various social determinants of zero-sanitation in order to achieve SDG 6.2 by 2030.

## Additional material


Online Supplementary Document
