# Supplementary material for: Prevalence of zero-sanitation in India: Patterns of change across the states and Union Territories, 1993-2021
Source: J Glob Health. 2023 Jul 28;13:04082. doi: 10.7189/jogh.13.04082 (PMC10373110; doi:10.7189/jogh.13.04082)
Supplement: Online Supplementary Document [file jogh-13-04082-s001.pdf]

Supplementary Table 1: The prevalence of Zero-Sanitation and 95% confidence interval (CI) for India and 36 states/Union Territories, 1993-2021

	**1993**		**1999**		**2006**		**2016**		**2021**	
	%	95% CI	%	95% CI	%	95% CI	%	95% CI	%	95% CI
**All India**	70.3	(70.2,70.5)	64.1	(63.9,64.2)	56.2	(56.0,56.3)	39.8	(39.8,39.9)	17.8	(17.7-17.9)
**States**										
Andhra Pradesh	78.1	(77.6-78.6)	75.4	(74.9-75.9)	56.9	(56.4-57.4)	38.4	(38.1-38.7)	14.9	(14.8-15.2)
Arunachal Pradesh	27.7	(23.9-31.6)	25.8	(22.2-29.5)	18.7	(15.5-21.9)	9.1	(7.9-10.3)	1.3	(0.8-1.8)
Assam	48.0	(47.1-48.8)	35.5	(34.7-36.4)	23.2	(22.5-23.9)	10.7	(10.4-10.9)	3.9	(3.8-4.1)
Bihar	82.4	(82.1-82.7)	80.9	(80.5-81.3)	73.2	(72.8-73.6)	65.9	(65.7-66.1)	37.7	(37.5-37.9)
Chhattisgarh	86.2	(85.6-86.9)	85.2	(84.5-85.9)	80.9	(80.2-81.6)	57.6	(57.2-58.0)	13.3	(13.0-13.5)
Goa	54.9	(51.0-58.9)	44.5	(40.7-48.3)	24.5	(21.3-27.6)	11.2	(10.1-12.3)	2.9	(2.5-3.5)
Gujarat	64.6	(63.9-65.2)	56.0	(55.4-56.6)	46.2	(45.6-46.9)	30.4	(30.2-30.6)	18.8	(18.6-18.9)
Haryana	73.9	(73.1-74.7)	62.7	(61.7-63.6)	48.7	(47.7-49.6)	10.2	(9.9-10.4)	2.9	(2.8-3.1)
Himachal Pradesh	88.9	(87.8-90.0)	74.3	(72.7-75.9)	55.6	(53.8-57.4)	14.5	(13.9-15.1)	6.8	(6.4-7.2)
Jharkhand	81.4	(80.5-82.2)	83.8	(83.1-84.4)	77.5	(76.7-78.2)	69.9	(69.6-70.2)	30.7	(30.3-30.9)
Karnataka	70.2	(69.7-70.8)	63.5	(62.9-64.1)	55.3	(54.7-55.9)	35.3	(35.0-35.5)	17.6	(17.4-17.8)
Kerala	28.5	(27.8-29.2)	13.7	(13.2-14.2)	3.4	(3.13-3.75)	0.7	(0.6-0.7)	0.1	(0.07-0.12)
Madhya Pradesh	75.9	(75.5-76.4)	74.9	(74.4-75.3)	73.1	(72.6-73.5)	57.1	(56.9-57.4)	23.1	(22.9-23.3)
Maharashtra	59.1	(58.7-59.6)	54.4	(53.9-54.8)	46.4	(45.9-46.8)	29.4	(29.2-29.5)	12.8	(12.7-12.9)
Manipur	16.0	(13.9-18.1)	8.0	(6.5-9.6)	4.5	(3.3-5.8)	1.2	(0.9-1.5)	0.2	(0.1-0.3)
Meghalaya	45.5	(42.6-48.4)	44.5	(41.6-47.5)	30.1	(27.7-32.5)	7.7	(7.1-8.4)	3.7	(3.2-4.1)
Mizoram	1.4	(2.8-2.5)	2.4	(0.9-3.8)	2.0	(0.7-3.3)	0.8	(0.4-1.2)	0.1	(-0.03-0.2)
Nagaland	20.5	(17.5-23.4)	24.3	(21.4-27.1)	14.7	(12.1-17.2)	1.5	(1.1-1.9)	0.4	(0.2-0.6)
Odisha	87.0	(86.5-87.5)	85.5	(85.0-86.1)	79.2	(78.6-79.8)	64.9	(64.6-65.2)	29.6	(29.3-29.9)
Punjab	62.6	(61.8-63.5)	48.2	(47.3-49.1)	28.7	(27.9-29.5)	7.0	(6.8-7.2)	2.5	(2.3-2.6)
Rajasthan	81.8	(81.3-82.2)	71.5	(70.9-72.1)	71.7	(71.2-72.2)	45.8	(45.5-46.0)	20.9	(20.7-21.1)
Sikkim			28.9	(23.3-34.6)	11.2	(7.7-14.7)	0.2	(-0.1-0.5)	0.2	(-0.04-0.5)
Tamil Nadu	69.2	(68.6-69.7)	64.8	(64.3-65.4)	56.5	(55.9-57.1)	38.3	(38.1-38.5)	18.4	(18.2-18.6)
Telangana	69.2	(68.4-69.9)	65.9	(65.2-66.6)	56.9	(56.4-57.4)	30.0	(29.7-30.3)	10.8	(10.6-11.1)
Tripura	19.1	(17.1-20.9)	9.1	(7.8-10.5)	3.1	(2.3-3.9)	2.2	(1.9-2.5)	0.9	(0.7-1.2)
Uttar Pradesh	79.0	(78.7-79.3)	73.6	(73.3-73.9)	66.5	(66.2-66.8)	53.9	(53.8-54.1)	21.1	(20.9-21.2)
Uttarakhand	65.9	(64.5-67.3)	63.4	(62.1-64.8)	43.2	(41.7-44.7)	17.2	(16.7-17.7)	5.9	(5.7-6.3)
West Bengal	61.5	(61.0-62.0)	55.9	(55.41-56.4)	41.7	(41.2-42.1)	25.0	(24.8-25.2)	11.1	(10.9-11.2)
**Union Territories**										
Andaman & Nicobar Islands							14.8	(12.3-17.2)	3.4	(2.1-4.6)
Chandigarh							1.8	(1.3-2.4)	1.4	(0.9-1.9)
Dadra & Nagar Haveli and Daman & Diu							33.2	(30.5-35.9)	9.9	(8.2-11.8)
Jammu & Kashmir	82.7	(81.1-84.3)	44.8	(43.3-46.2)	35.8	(34.5-37.2)	21.2	(20.7-21.7)	5.5	(5.2-5.7)
Ladakh					35.8	(34.5-37.2)	1.1	(0.1-2.1)	0.5	(-0.2-1.2)
Lakshadweep									0.0	
Delhi	13.2	(12.3-14.1)	5.6	(5.0-6.1)	7.3	(6.7-7.9)	3.7	(3.6-3.9)	0.6	(0.5-0.7)
Puducherry							31.2	(29.5-32.9)	8.7	(7.6-9.8)

Note: The colors represent the following categories of the prevalence of Zero-Sanitation (%):

<20%	>=20 AND <40	>=40 AND <60	>=60 AND <80	>=80

Supplementary table 2: Progress towards SDG 6.2 by state and Union Territory

	Required change per year	2016-2021 actual change per year	Will meet SDG 6.2 by 2030
**All India**	-2.8	-4.4	Yes
**States**			
Andhra Pradesh	-2.7	-4.7	Yes
Arunachal Pradesh	-0.7	-1.6	Yes
Assam	-0.8	-1.4	Yes
Bihar	-4.7	-5.6	Yes
Chhattisgarh	-4.1	-8.9	Yes
Goa	-0.8	-1.7	Yes
Gujarat	-2.2	-2.3	No
Haryana	-0.7	-1.5	Yes
Himachal Pradesh	-1.0	-1.5	Yes
Jharkhand	-5.0	-7.8	Yes
Karnataka	-2.5	-3.5	Yes
Kerala	0.0	-0.1	Yes
Madhya Pradesh	-4.1	-6.8	Yes
Maharashtra	-2.1	-3.3	Yes
Manipur	-0.1	-0.2	Yes
Meghalaya	-0.6	-0.8	Yes
Mizoram	-0.1	-0.1	Yes
Nagaland	-0.1	-0.2	Yes
Odisha	-4.6	-7.1	Yes
Punjab	-0.5	-0.9	Yes
Rajasthan	-3.3	-5.0	Yes
Sikkim	0.0	0.0	No
Tamil Nadu	-2.7	-4.0	Yes
Telangana	-2.1	-3.8	Yes
Tripura	-0.2	-0.3	Yes
Uttar Pradesh	-3.9	-6.6	Yes
Uttarakhand	-1.2	-2.3	Yes
West Bengal	-1.8	-2.8	Yes
**Union Territories**			
Andaman & Nicobar Islands	-1.1	-2.3	Yes
Chandigarh	-0.1	-0.1	No
Dadra & Nagar Haveli and Daman & Diu	-2.4	-4.7	Yes
Jammu & Kashmir	-1.5	-3.1	Yes
Ladakh	-0.1	-0.1	Yes
Lakshadweep	0.0	0.0	NA
Delhi	-0.3	-0.6	Yes
Puducherry	-2.2	-4.5	Yes

Supplementary table 3: The prevalence of Zero-Sanitation and 95% confidence interval (CI) in urban communities for India and 36 states/Union Territories, 1993-2021

	1993		1999		2006		2016		2021.0	
**All India**	23.9	(23.7-24.2)	19.1	(18.86-19.28)	16.9	(16.8-17.1)	10.6	(10.6-10.7)	4.4	(4.4-4.5)
**States**										
Andhra Pradesh	38.6	(37.3-39.9)	38.4	(37.1-39.6)	24.0	(23.3-24.8)	11.0	(10.6-11.3)	3.5	(3.3-3.7)
Arunachal Pradesh	12.8	(4.4-21.1)	2.1	(-1.2-5.4)	5.1	(1.5-8.7)	1.0	(0.2-1.9)	0.3	(-0.3-0.9)
Assam	7.2	(5.9-8.5)	4.4	(3.1-5.7)	2.5	(1.9-3.1)	1.0	(0.8-1.2)	0.9	(0.7-1.1)
Bihar	31.6	(30.5-32.7)	30.4	(28.9-31.9)	27.8	(26.7-28.8)	22.0	(21.5-22.5)	11.2	(10.9-11.5)
Chhattisgarh	31.9	(29.7-34.0)	56.2	(53.9-58.5)	32.3	(30.4-34.2)	16.9	(16.3-17.5)	3.1	(2.8-3.4)
Goa	37.2	(31.7-42.7)	31.3	(25.7-36.9)	14.9	(11.3-18.4)	9.7	(8.5-11.0)	1.2	(0.8-1.7)
Gujarat	28.2	(27.2-29.2)	22.6	(21.7-23.4)	12.0	(11.4-12.6)	6.0	(5.8-6.2)	2.9	(2.8-3.1)
Haryana	28.3	(26.7-30.0)	17.7	(16.3-19.1)	12.0	(10.8-13.2)	3.5	(3.3-3.7)	1.0	(0.9-1.1)
Himachal Pradesh	23.5	(18.3-28.6)	15.7	(11.4-20.1)	9.7	(6.4-12.9)	4.6	(3.5-5.7)	2.7	(2.0-3.5)
Jharkhand	35.1	(32.6-37.5)	32.2	(30.3-34.1)	25.5	(24.1-26.9)	25.8	(25.1-26.4)	9.7	(9.2-10.1)
Karnataka	26.9	(25.9-27.9)	20.3	(19.5-21.1)	19.4	(18.7-20.2)	11.8	(11.5-12.1)	7.3	(7.1-7.5)
Kerala	15.5	(14.2-16.6)	6.5	(5.7-7.3)	1.4	(1.0-1.7)	0.2	(0.2-0.3)	0.0	(0.0-0.0)
Madhya Pradesh	27.7	(26.7-28.6)	30.2	(29.2-31.2)	28.2	(27.2-29.1)	16.1	(15.8-16.5)	6.4	(6.1-6.6)
Maharashtra	16.9	(16.3-17.4)	14.4	(13.9-14.9)	12.6	(12.1-12.9)	10.1	(9.9-10.3)	3.3	(3.2-3.4)
Manipur	5.3	(3.1-7.6)	0.3	(-0.3-0.9)	0.5	(-0.2-1.3)	0.5	(0.2-0.8)	0.2	(-0.02-0.3)
Meghalaya	3.3	(1.1-5.6)	1.3	(-0.2-2.8)	1.6	(0.2-2.9)	0.5	(0.1-0.9)	0.3	(0.01-0.6)
Mizoram	0.4	(-0.4-1.3)	0.3	(-0.4-0.9)			0.0	(-0.0-0.6)		
Nagaland	5.2	(1.7-8.7)	1.8	(-0.2-3.8)	0.7	(-0.4-1.8)	0.3	(-0.0-0.6)	0.3	(-0.1-0.6)
Odisha	47.6	(45.7-49.5)	43.3	(41.2-45.5)	39.2	(37.5-40.9)	29.5	(28.8-30.2)	16.9	(16.4-17.6)
Punjab	25.4	(23.9-26.8)	7.4	(6.6-8.3)	5.7	(5.0-6.4)	2.0	(1.8-2.2)	1.3	(1.2-1.4)
Rajasthan	33.6	(32.3-34.9)	22.0	(20.9-22.9)	16.4	(15.6-17.2)	11.2	(10.9-11.5)	2.6	(2.4-2.7)
Sikkim			11.8	(0.5-23.1)	0.2	(-1.0-1.5)	0.1	(-0.2-0.5)	0.2	(-0.2-0.6)
Tamil Nadu	28.9	(28.0-29.9)	24.5	(23.7-25.3)	26.3	(25.6-27.1)	16.6	(16.4-16.9)	7.6	(7.4-7.8)
Telangana	12.9	(11.8-13.9)	11.4	(10.5-12.3)	24.0	(23.3-24.8)	6.0	(5.7-6.2)	2.1	(1.9-2.3)
Tripura	1.1	(-0.1-2.2)	1.8	(0.5-3.2)			0.5	(0.2-0.8)	0.2	(0.0-0.3)
Uttar Pradesh	21.9	(21.3-22.5)	16.1	(15.6-16.7)	15.7	(15.2-16.2)	10.3	(10.1-10.5)	5.0	(4.9-5.2)
Uttarakhand	10.6	(9.0-12.2)	14.3	(12.2-16.4)	5.8	(4.4-7.1)	3.0	(2.6-3.4)	2.0	(1.7-2.3)
West Bengal	19.2	(18.5-19.9)	9.7	(9.2-10.3)	10.9	(10.4-11.5)	10.3	(10.1-10.6)	2.9	(2.7-3.0)
**Union Territories**										
Andaman & Nicobar Islands							2.5	(0.8-4.1)	2.7	(-0.3-0.8)
Chandigarh							1.9	(1.3-2.5)	1.4	(0.9-1.9)
Dadra & Nagar Haveli and Daman & Diu							5.3	(3.5-7.0)	2.2	(0.9-3.5)
Jammu & Kashmir	23.2	(18.8-27.5)	10.1	(8.2-12.0)	12.7	(10.9-14.4)	3.4	(2.9-3.8)	0.6	(0.4-0.8)
Ladakh					12.7	(10.9-14.4)				
Lakshadweep										
Delhi	10.8	(9.9-11.7)	3.6	(3.2-4.1)	5.5	(4.9-6.1)	3.8	(3.6-3.9)	0.6	(0.5-0.7)
Puducherry							22.0	(20.1-23.9)	3.4	(2.5-4.2)

Note: The colors represent the following categories of the prevalence of Zero-Sanitation (%):

<20%	>=20 AND <40	>=40 AND <60	>=60 AND <80	>=80

Supplementary table 4: The prevalence of Zero-Sanitation and 95% confidence interval (CI) in rural communities for India and 36 states/Union Territories-1993-2021

	**1993**		**1999**		**2006**		**2016**		**2021**	
**All India**	86.8	(86.7-86.9)	80.3	(80.2-80.4)	73.8	(73.7-73.9)	54.3	(54.2-54.4)	24.1	(24.0-24.1)
**States**										
Andhra Pradesh	91.4	(91.0-91.9)	86.6	(86.1-87.1)	73.2	(72.7-73.8)	50.4	(50.1-50.8)	20.1	(19.8-20.4)
Arunachal Pradesh	29.8	(25.6-33.9)	29.4	(25.3-33.5)	23.3	(19.2-27.3)	11.7	(10.2-13.2)	1.5	(0.9-2.0)
Assam	53.4	(52.5-54.3)	38.2	(37.3-39.1)	27.5	(26.7-28.3)	12.2	(11.9-12.5)	4.5	(4.3-4.6)
Bihar	91.3	(90.9-91.5)	85.8	(85.4-86.1)	81.8	(81.4-82.2)	72.3	(72.1-72.5)	42.7	(42.5-42.9)
Chhattisgarh	97.4	(97.0-97.7)	91.8	(91.2-92.4)	94.0	(93.6-94.5)	69.8	(69.4-70.2)	16.2	(15.9-16.5)
Goa	71.1	(66.1-76.0)	53.3	(48.4-58.3)	36.6	(31.3-42.0)	13.6	(11.7-15.5)	5.6	(4.4-6.7)
Gujarat	83.8	(83.2-84.3)	79.4	(78.7-80.1)	70.4	(69.7-71.1)	49.1	(48.7-49.4)	30.1	(29.8-30.5)
Haryana	90.0	(89.4-90.7)	80.8	(79.9-81.7)	63.2	(62.1-64.3)	14.4	(14.1-14.8)	3.9	(3.8-4.2)
Himachal Pradesh	95.4	(94.6-96.2)	80.2	(78.7-81.7)	61.0	(59.1-62.9)	15.6	(14.9-16.2)	7.4	(6.9-7.8)
Jharkhand	92.2	(91.5-92.8)	95.8	(95.4-96.2)	95.0	(94.6-95.4)	84.5	(84.2-84.8)	37.3	(36.9-37.7)
Karnataka	90.8	(90.4-91.2)	86.5	(86.0-87.0)	78.4	(77.8-79.0)	52.4	(52.1-52.8)	24.4	(24.1-24.6)
Kerala	33.5	(32.7-34.4)	15.9	(15.3-16.6)	4.5	(4.1-4.9)	1.1	(0.9-1.2)	0.2	(0.1-0.2)
Madhya Pradesh	93.4	(93.0-93.7)	92.1	(91.7-92.4)	89.3	(88.9-89.7)	73.9	(73.7-74.1)	29.4	(29.1-29.7)
Maharashtra	89.9	(89.5-90.2)	84.4	(83.9-84.8)	78.1	(77.6-78.6)	46.6	(46.4-46.9)	20.5	(20.3-20.7)
Manipur	21.1	(18.3-23.9)	11.8	(9.5-14.1)	6.4	(4.6-8.2)	1.6	(1.2-2.1)	0.3	(0.1-05)
Meghalaya	56.9	(53.6-60.1)	55.3	(52.1-58.6)	38.9	(35.9-41.8)	9.5	(8.7-10.3)	4.5	(3.9-5.1)
Mizoram	2.5	(0.3-4.7)	4.7	(1.9-7.6)	4.1	(1.4-6.8)	1.8	(0.9-2.6)	0.2	(-0.1-0.5)
Nagaland	24.5	(20.9-27.9)	29.9	(26.5-33.3)	19.6	(16.3-22.9)	2.2	(1.6-2.8)	0.4	(0.1-0.7)
Odisha	94.2	(93.8-94.6)	90.8	(90.4-91.3)	87.3	(86.8-87.8)	72.1	(71.7-72.4)	32.3	(32.0-32.7)
Punjab	77.3	(76.4-78.2)	65.6	(64.6-66.7)	42.0	(40.9-43.1)	10.2	(9.9-10.5)	3.2	(2.9-3.3)
Rajasthan	93.0	(92.7-93.3)	87.8	(87.4-88.3)	91.6	(91.2-91.9)	57.0	(56.7-57.3)	26.7	(26.4-26.9)
Sikkim			31.5	(25.3-37.7)	13.7	(9.5-17.9)	0.3	(-0.1-0.7)	0.3	(-0.1-0.6)
Tamil Nadu	91.0	(90.6-91.4)	86.2	(85.7-86.7)	82.8	(82.2-83.5)	60.3	(60.0-60.6)	28.6	(28.3-28.9)
Telangana	92.9	(92.4-93.5)	89.3	(88.7-89.8)	73.2	(72.7-73.8)	51.3	(50.8-51.8)	16.1	(15.7-16.4)
Tripura	23.1	(20.8-25.4)	10.8	(9.2-12.4)	3.6	(2.7-4.6)	2.9	(2.5-3.3)	1.3	(0.9-1.6)
Uttar Pradesh	92.8	(92.6-93.0)	88.5	(88.2-88.7)	82.8	(82.5-83.1)	68.5	(68.3-68.7)	26.4	(26.2-26.5)
Uttarakhand	91.5	(90.6-92.5)	77.7	(76.4-79.0)	57.5	(55.7-59.2)	24.9	(24.2-25.6)	7.9	(7.4-8.3)
West Bengal	79.6	(79.2-80.1)	71.3	(70.8-71.8)	54.5	(53.9-55.1)	32.0	(31.7-32.2)	15.0	(14.8-15.2)
**Union Territories**										
Andaman & Nicobar Islands							23.8	(19.9-27.7)	5.2	(3.2-7.2)
Chandigarh							0.9	(-1.2-3.1)		
Dadra & Nagar Haveli and Daman & Diu							66.7	(62.7-70.7)	16.9	(13.8-19.9)
Jammu & Kashmir	94.6	(93.5-95.6)	53.6	(52.0-55.2)	44.5	(42.8-46.1)	28.7	(28.0-29.3)	7.2	(6.8-7.5)
Ladakh					44.5	(42.8-46.1)	1.5	(1.3-2.8)	0.6	(-0.2-1.4)
Lakshadweep										
Delhi	45.0	(39.9-50.1)	27.9	(24.1-31.7)	27.7	(23.8-31.6)	1.9	(0.5-3.4)	2.0	(1.1-2.9)
Puducherry							51.7	(48.3-55.1)	20.9	(18.0-23.8)

Note: The colors represent the following categories of the prevalence of Zero-Sanitation (%):

<20%	>=20 AND <40	>=40 AND <60	>=60 AND <80	>=80

Supplementary figure 1: Status of States on the prevalence of Zero-Sanitation from 1993 to 2021

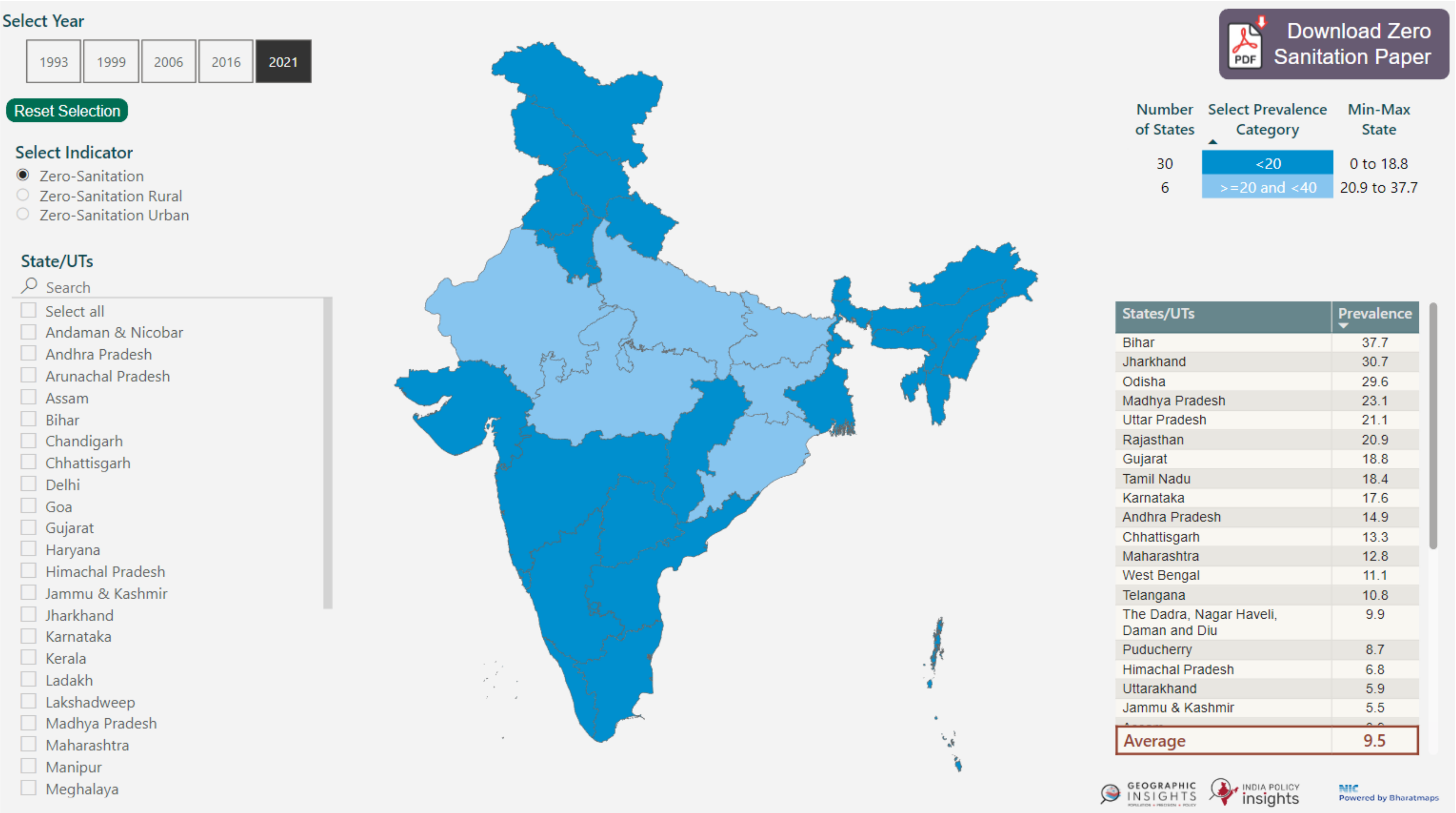

Note: See https://geographicinsights.iq.harvard.edu/State-Zero-Sanitation
